# Supplementary material for: Age and sex influence diurnal memory oscillations, circadian rhythmicity, and Per1 expression
Source: Biol Sex Differ. 2025 Oct 14;16:74. doi: 10.1186/s13293-025-00756-x (PMC12522461; doi:10.1186/s13293-025-00756-x)
Supplement: Supplementary file 1 — Supplementary Material 1 [file 13293_2025_756_MOESM1_ESM.pdf]

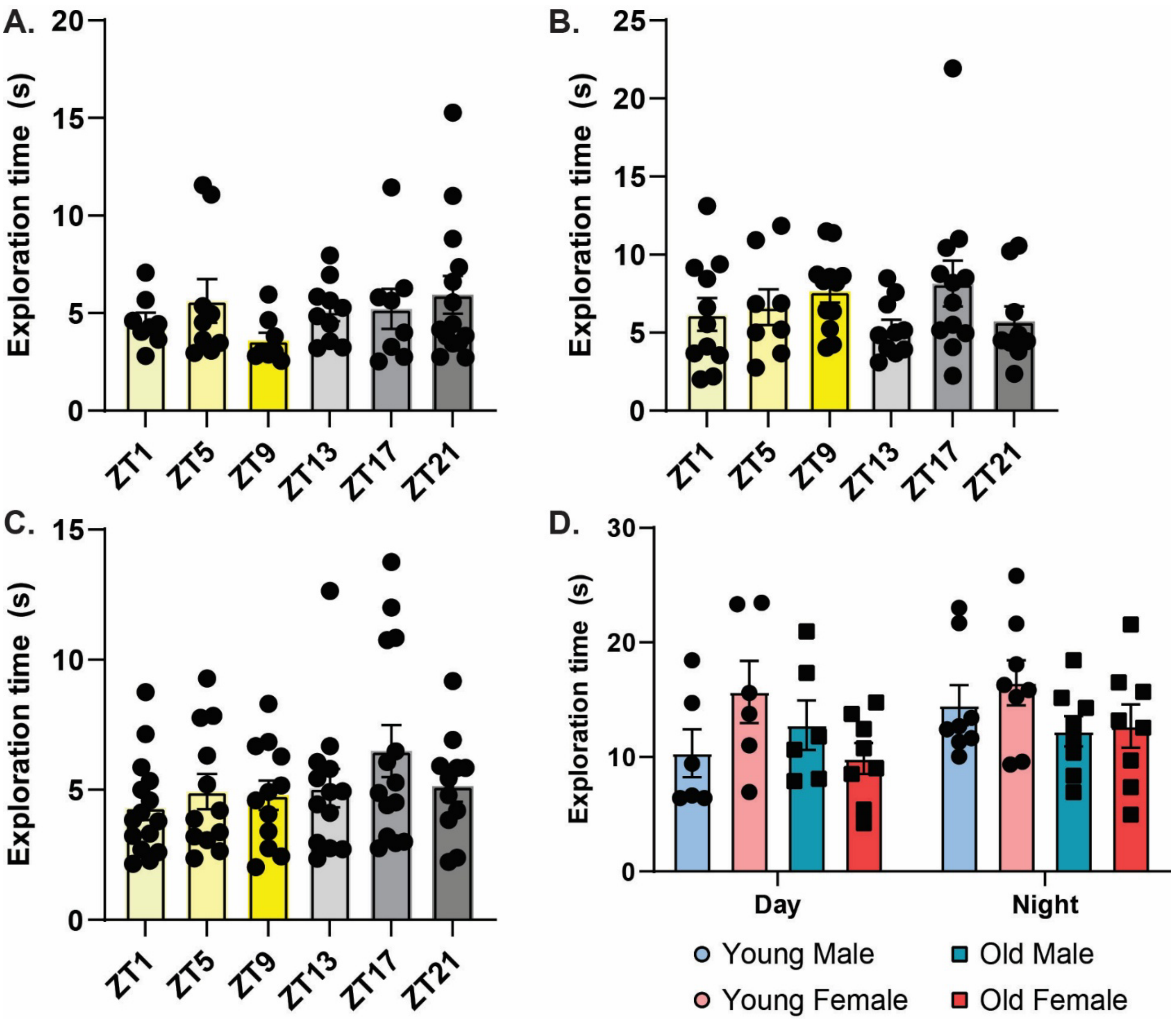

Supplemental Figure 1. Total exploration does not differ across the diurnal cycle. **A.** Total object exploration during the test session for the young female circadian memory experiment (Fig. 1C-D; n=8-14/timepoint). **B.** Total object exploration during the test session for the old female circadian memory experiment (Fig. 1E-F; n=8-12/timepoint). **C.** Total object exploration during the test session for the old male circadian memory experiment (Fig. 2; n=11-15/timepoint). **D.** Total object exploration during the training session for the *Per1* induction experiment Fig. 3; 6-8/timepoint). ZT = Zeitgeber Time, where ZT0 = 6am (7am DST), lights on, ZT12 = 6pm (7pm DST), lights off.
