# Supplementary material for: Age and sex influence diurnal memory oscillations, circadian rhythmicity, and Per1 expression
Source: Biol Sex Differ. 2025 Oct 14;16:74. doi: 10.1186/s13293-025-00756-x (PMC12522461; doi:10.1186/s13293-025-00756-x)
Supplement: Supplementary file 2 — Supplementary Material 2 [file 13293_2025_756_MOESM2_ESM.pdf]

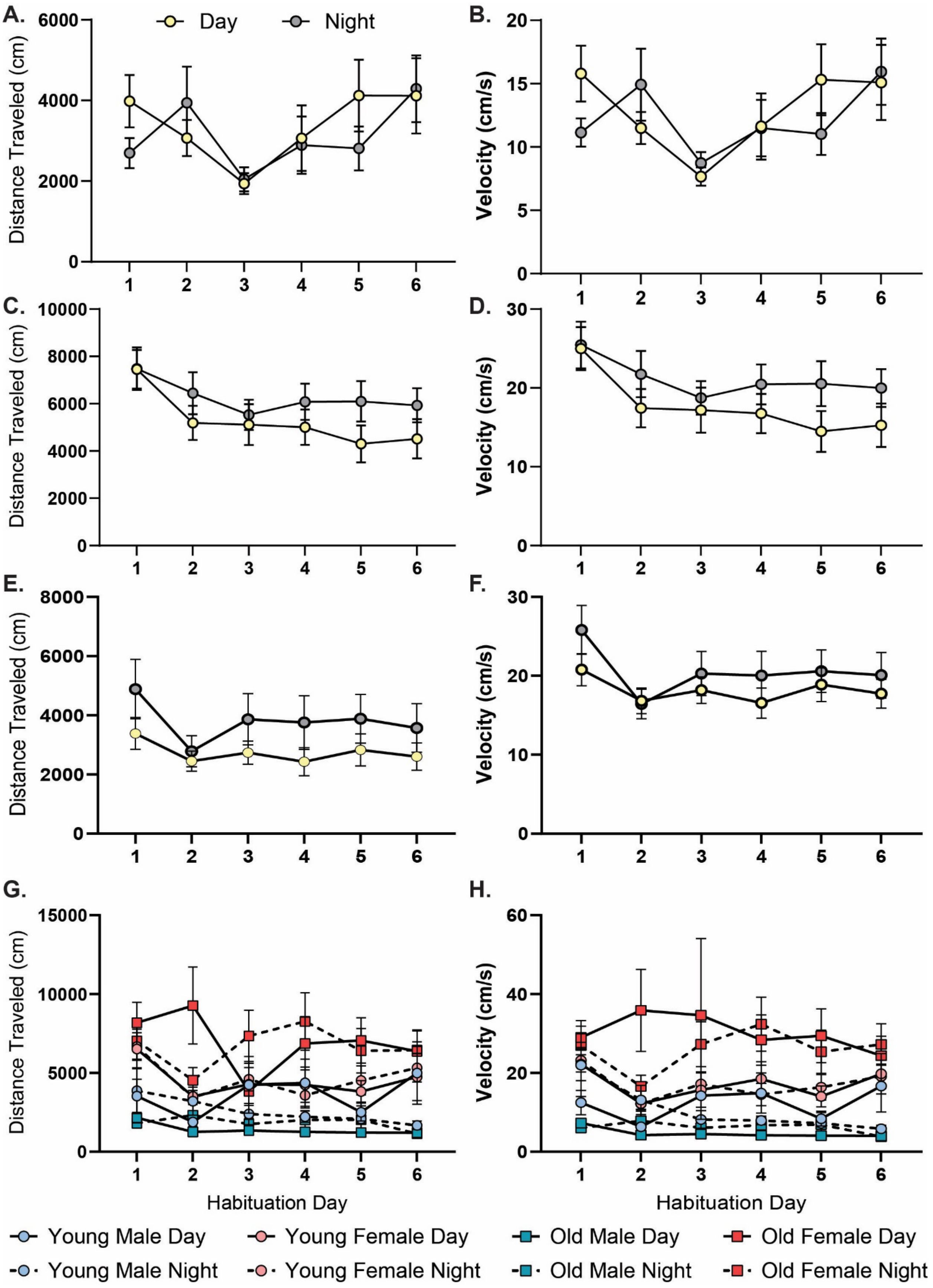
A.
B.
C.
D.
E.
F.
G.
H.
Day
Night
Distance Traveled (cm)
Velocity (cm/s)
0
2000
4000
6000
8000
10000
15000
5000
5
10
15
20
30
40
60
1
2
3
4
5
6
Habituation Day
Young Male Day
Young Female Day
Old Male Day
Old Female Day
Young Male Night
Young Female Night
Old Male Night
Old Female Night

Supplemental Figure 2. Mouse distance traveled and velocity is similar across the diurnal cycle, with no effect of age but a sex effect, with females showing more movement during habituation. **A.** Distance traveled (cm) and **B.** velocity (cm/s) of young female mice habituated during the day (yellow symbols; ZT1, ZT5, ZT9) and at night (grey symbols; ZT13, ZT17, ZT21) across the 6 habituation days from the young female circadian experiment (Fig. 1C-D; n=25-32/timepoint). **C.** Distance traveled (cm) and **D.** velocity (cm/s) of old female mice habituated during the day and at night across the 6 habituation days from the old female circadian experiment (Fig. 1E-F; n=31/timepoint). **E.** Distance traveled (cm) and **F.** velocity (cm/s) of old male mice habituated during the day and at night across the 6 habituation days from the old male circadian experiment (Fig. 2; n=38-39/timepoint). **G.** Distance traveled (cm) and **H.** velocity (cm/s) across the 6 habituation days from the *Per1* induction experiment (Fig. 3; n=12-17/cohort).
