# Supplementary material for: Age and sex influence diurnal memory oscillations, circadian rhythmicity, and Per1 expression
Source: Biol Sex Differ. 2025 Oct 14;16:74. doi: 10.1186/s13293-025-00756-x (PMC12522461; doi:10.1186/s13293-025-00756-x)
Supplement: Supplementary file 3 — Supplementary Material 3 [file 13293_2025_756_MOESM3_ESM.pdf]

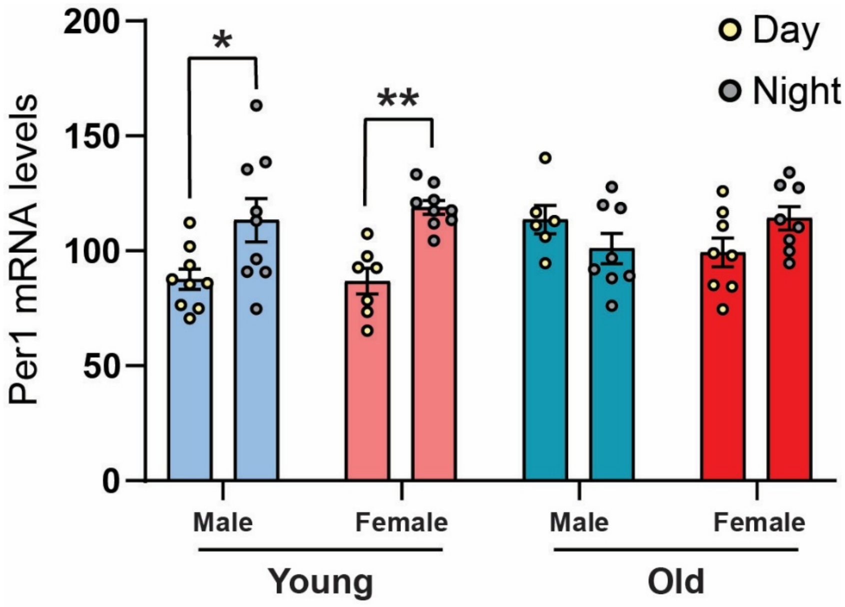

Supplemental Figure 3. *Per1* mRNA levels in homecage young male (n=9/cohort) and female (n=7-9/cohort) mice were significantly higher during the night compared to the day, but no differences were seen in old male (n=6-8/cohort) and female (n=8/cohort) mice. * = $p<0.05$, ** = $p<0.01$.
