# Supplementary material for: Age and sex influence diurnal memory oscillations, circadian rhythmicity, and Per1 expression
Source: Biol Sex Differ. 2025 Oct 14;16:74. doi: 10.1186/s13293-025-00756-x (PMC12522461; doi:10.1186/s13293-025-00756-x)
Supplement: Supplementary file 4 — Supplementary Material 4 [file 13293_2025_756_MOESM4_ESM.pdf]

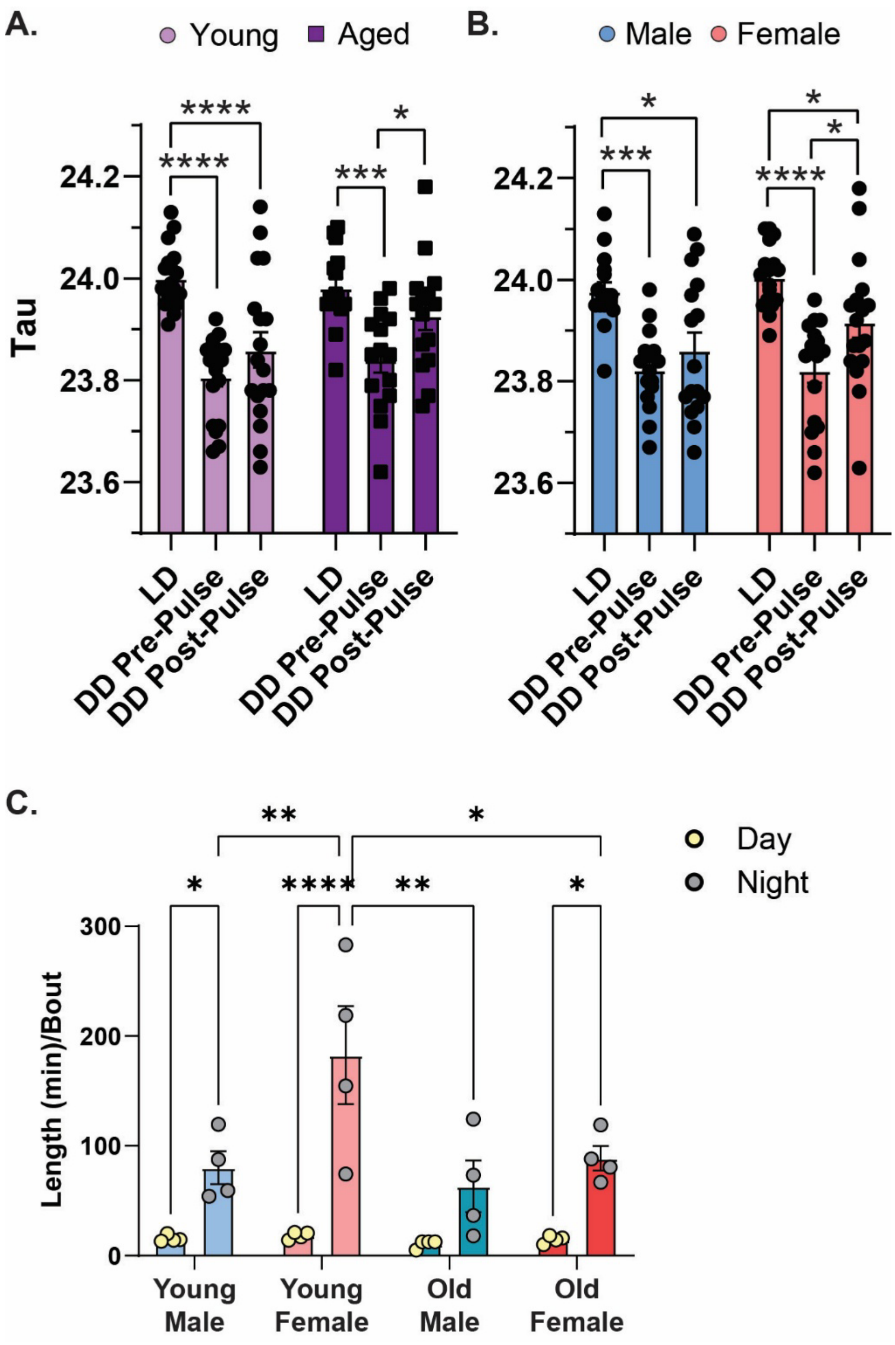

Supplemental Figure 4. Circadian period and activity as compared by sex and age. **A.** There is no effect of age on the circadian period length (free-running tau; n=15-18/cohort). **B.** There is also no effect of sex on free-running tau (n=16-17/cohort) **C.** The length (in minutes) of bouts during the dark phase (grey) is significantly higher in young males, young females, and old females than the light phase (yellow) while old males show no difference (n=8-9/cohort). LD = light/dark, DD = dark/dark. ns = not significant, * = p<0.05, ** = p<0.01, *** = p<0.001, **** = p<0.0001 as compared between groups.
