# Supplementary material for: Age and sex influence diurnal memory oscillations, circadian rhythmicity, and Per1 expression
Source: Biol Sex Differ. 2025 Oct 14;16:74. doi: 10.1186/s13293-025-00756-x (PMC12522461; doi:10.1186/s13293-025-00756-x)
Supplement: Supplementary file 5 — Supplementary Material 5 [file 13293_2025_756_MOESM5_ESM.pdf]

A. B. C.

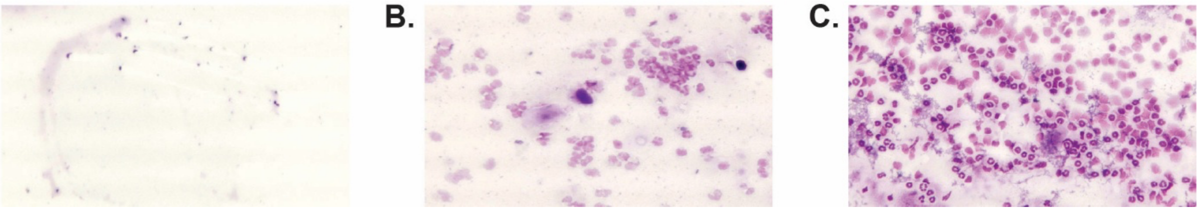

Supplemental Figure 5. Representative vaginal cytology smears from **A.** Acyclic 19-month-old mouse **B.** Acyclic Persistent Diestrus 19-month-old mouse **C.** Cyclic Diestrus 8-week-old mouse. Persistent Diestrus and Diestrus in cyclic mice is easy to differentiate due to the quantity of leukocytes present in the smear (smaller quantity for persistent diestrus).
